# Sinus venosus incorporation: contentious issues and operational criteria for developmental and evolutionary studies

**DOI:** 10.1111/joa.12962

**Published:** 2019-03-12

**Authors:** Jaeike W. Faber, Bastiaan J. Boukens, Roelof‐Jan Oostra, Antoon F. M. Moorman, Vincent M. Christoffels, Bjarke Jensen

**Affiliations:** ^1^ *Department of Medical Biology* *Amsterdam Cardiovascular Sciences* *Amsterdam UMC* *University of Amsterdam* *Amsterdam* The Netherlands

**Keywords:** development, evolution, heart, sinuatrial valve

## Abstract

The sinus venosus is a cardiac chamber upstream of the right atrium that harbours the dominant cardiac pacemaker. During human heart development, the sinus venosus becomes incorporated into the right atrium. However, from the literature it is not possible to deduce the characteristics and importance of this process of incorporation, due to inconsistent terminology and definitions in the description of multiple lines of evidence. We reviewed the literature regarding the incorporation of the sinus venosus and included novel electrophysiological data. Most mammals that have an incorporated sinus venosus show a loss of a functional valve guard of the superior caval vein together with a loss of the electrical sinuatrial delay between the sinus venosus and the right atrium. However, these processes are not necessarily intertwined and in a few species only the sinuatrial delay may be lost. Sinus venosus incorporation can be characterised as the loss of the sinuatrial delay of which the anatomical and molecular underpinnings are not yet understood.

## Introduction

The sinus venosus, or systemic venous sinus, is the cardiac chamber with myocardial walls located upstream of the right atrium in tetrapods and the single atrium in fish. It is the region that harbours the dominant cardiac pacemaker or sinus node (Carmona et al. 2018). In ectotherms the sinus venosus is known to assist the filling of the right atrium whereby it supports the cardiac output (Jensen et al. 2017). This function is lost in endotherms for reasons still unknown. In the embryonic mammalian heart, the sinus venosus is the confluence of the vitelline, cardinal and umbilical veins. These vessels later remodel to form both the right and left superior, or anterior, caval veins and the inferior, or posterior, caval vein, which is also located on the right side of the heart. On the border between the sinus venosus and the atrium, the sinuatrial valve is found (Carmona et al. 2018). Later in mammalian development, the sinus venosus is often described as being incorporated into the right atrium whereby it becomes the dorsal smooth wall of the right atrium, the so‐called sinus venarum (Keith & Flack, 1907; Carmona et al. 2018).

From the literature it is not possible to form a firm understanding of what the process of incorporation actually entails. In recent reviews it is stated that by incorporation, the sinus venosus becomes ‘the sinoatrial node in the atrial wall’ (Stephenson et al. 2017) whereas other authors state that incorporation is revealed by the appearance of the sinuatrial valve (Gittenberger‐De Groot et al. 2005), even though the sinuatrial valve forms earlier than the anatomically recognisable sinus node (Sizarov et al. 2010). Widely used textbooks mention and illustrate incorporation of the sinus venosus (Sadler, 2006; Moore et al. 2008; Schoenwolf et al. 2009; Standring et al. 2016). Between textbooks, however, the resultant sinus venarum differs in the extent and timing of its appearance and this likely reflects the absence of criteria and definitions for incorporation that are commonly agreed on. The situation is exacerbated by differences in terminology. The process of incorporation has also been described as merger (Keith & Flack, 1907; Carmona et al. 2018), assimilation (D'Cruz & Smith, 1995) and atrialisation (Van Weerd & Christoffels, 2016; Weisbrod et al. 2016). These terms connote a change in anatomy and/or a change in gene and protein expression, or even changes in the electrical activation of the sinus myocardium compared to that of the atrial myocardium (Jensen et al. 2014a). This inconsistency in the literature may have resulted from the use of multiple lines of evidence that range from studies on cardiac development (Steding et al. 1990; Gittenberger‐De Groot et al. 2005), comparative cardiac anatomy (Keith & Flack, 1907; Benninghoff, 1933) and electrophysiological properties of the tissue (Jensen et al. 2014a, 2017), to gene and protein expression profiles (Mommersteeg et al. 2007; Sizarov et al. 2010). Often, it is not stated which line of evidence has been used to declare the process of incorporation as complete. The absence of quantifiable anatomical features only compounds the confusion. Whereas, as a comparative example, the extent of incorporation of the pulmonary venous pole into the left atrium during human development can be assessed by the number of venous orifices in the atrial wall (Blom et al. 2001), the sinus venosus incorporation into the right atrium is based solely on qualitative observations. Here, we review the literature and contribute novel anatomical and electrophysiological data to clarify what constitutes incorporation of the sinus venosus.

## Methods

### Literature search

The four lines of evidence that support incorporation of the sinus venosus, i.e. ontogeny, comparative anatomy, physiology and molecular markers, were distilled from the literature and summarised. Additionally, the terminology used to describe the incorporation process, as found in the literature, is also discussed.

### Anatomy

Two adult human hearts, one normal (specimen code S96‐71) and one with a persistent left superior caval vein (specimen code T77‐3569), were macroscopically assessed. The hearts were acquired from the collection at the Amsterdam University Medical Centres, at the Academic Medical Centre, Amsterdam. Additionally, we took new photos of a Gila monster heart (*Heloderma suspectum*), previously described in Jensen et al. (2013).

### *In situ* hybridisation

Hearts of a mice from embryonic day 14.5, neonatal day 1 and adult age (all *n* = 1) had previously been fixed in a 4% paraformaldehyde solution before being embedded in paraffin. Sections of 8 (in case of 14.5 days of gestation) or 12 μm thick had been made before *in situ* hybridisation with RNA probes against either a mix of αMHC, βMHC and cTnI (in case of embryonic day 14.5), cTnI (in case of the neonate) or *Nppa* (in the adult). Extended methods are described in Soufan et al. (2003), Hoogaars et al. (2007) and Aanhaanen et al. (2011).

### Electrophysiology

Embryonic day 14.5 and adult mouse hearts were investigated by optical mapping, after which activation patterns were reconstructed. An electrocardiogram (ECG) was performed on a neonatal mouse. The methods that were used have been described previously (Aanhaanen et al. 2011).

## Results

### Ontogeny

**Figure 1:**
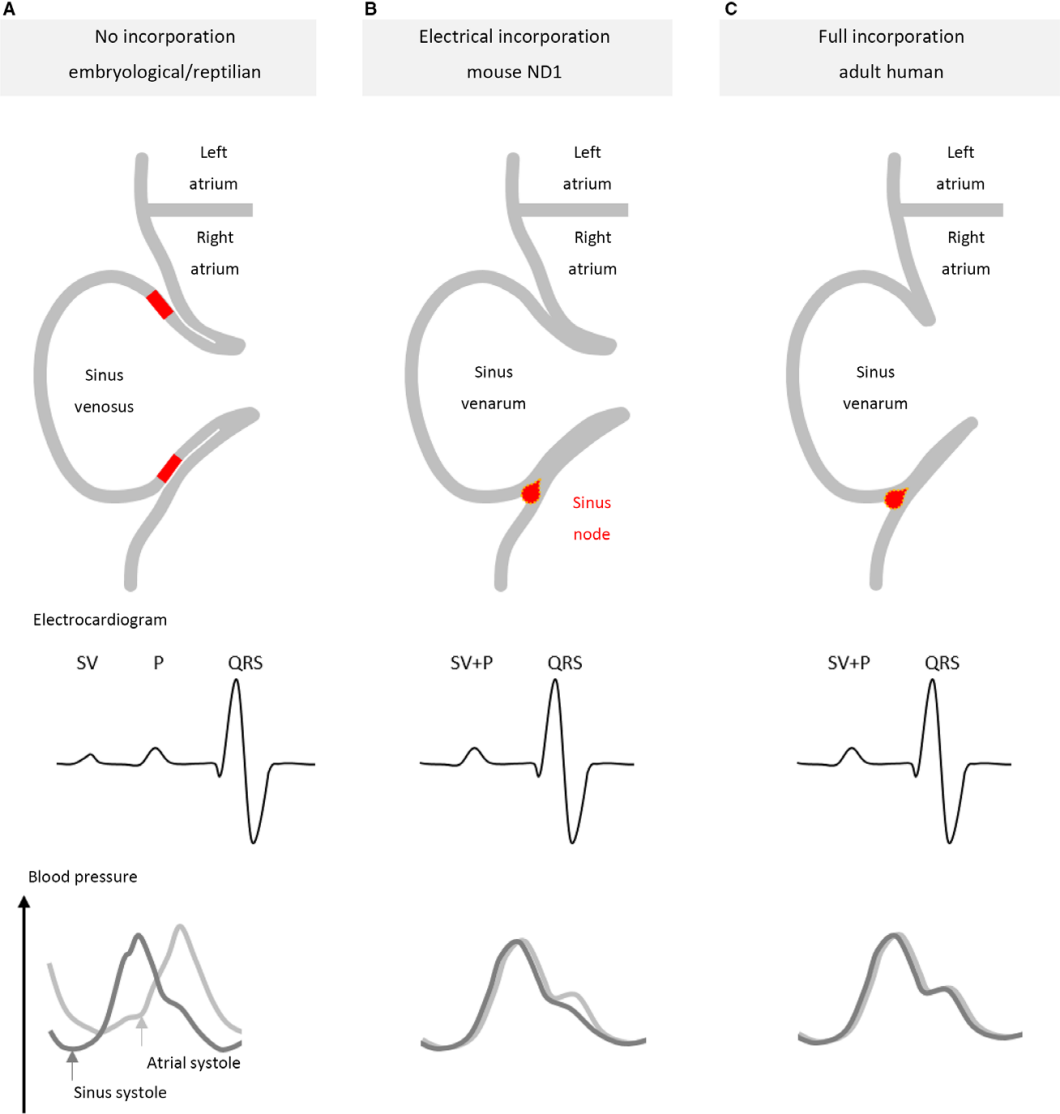
Gradations of sinus venosus incorporation into the right atrium. The region of the dominant pacemaker is schematised in red. (A) In the embryological and reptilian settings, there is no anatomical and no electrical incorporation. This is reflected in the early deflection of the sinus venosus (SV) on the electrocardiogram as compared to the atrial deflection (P) and in the differences in blood pressure in the sinus venosus (dark grey) and the right atrium (light grey). The onset of the sinus systole and atrial systole are marked with arrows in the blood pressure trace and coincides with the SV and P deflections, respectively, on the electrocardiogram. (B) In the mouse at neonatal day 1 there is no anatomical but only an electrical incorporation. The SV‐wave in the electrocardiogram is lost, and the systole of the sinus venosus and the right atrium coincide. Nonetheless, higher blood pressures in the right atrium can be envisioned to have a limited impact on the blood pressures in the sinus venarum due to the presence of a competent sinuatrial valve. (C) In the fully incorporated sinus venosus, in addition to the electrical incorporation, the competence of the sinuatrial valve is lost, resulting in a similar blood pressure during systole in the sinus venosus and the right atrium.

The key features of sinus venosus incorporation in development are schematised in Fig. 1. In most adult vertebrates, three caval veins connect to the atrium (Keith & Flack, 1907; Benninghoff, 1933). Only in humans and some other mammals does the left superior caval vein regress during normal foetal development to form the coronary sinus (Webb et al. 2001). Myocardium develops in the walls of the future right superior caval vein, future left superior caval vein or coronary sinus, and in some species, in the future inferior caval vein. Mouse studies revealed that this myocardium develops from a population of *Tbx18*‐positive mesenchyme that, in contrast to the rest of the cardiac myocardium, does not express *Nkx2‐5* (Christoffels et al. 2006). Because of the presence of myocardium, these parts, which will later become the caval veins despite the persistence of the myocardium, are also referred to as sinus horns (Keith & Flack, 1907). The boundary of the sinus venosus with the veins is drawn at the border of this myocardium. We propose that this border of the venous wall with the myocardial wall is applicable to all stages of ontogeny. In parallel, the sinuatrial valve between the sinus venosus and atrium becomes prominent (Steding et al. 1990). Its leaflets comprise two layers of myocardium, one from the sinus venosus, expressing sinus venosus marker genes, and one from the atrium, expressing atrial genes (Wessels et al. 1990; Mommersteeg et al. 2007). A similar layered build‐up of the sinuatrial leaflets is also seen in fish, reptiles and birds (Keith & Flack, 1907; Adams, 1937; Gallego et al. 1997; Jensen et al. 2017). In the early embryonic human and mouse, all sinus horns lie upstream of the single, prominent sinuatrial valve and the sinus venosus is not considered to be incorporated (Benninghoff, 1933; Steding et al. 1990) (Fig. 1A).

In the adult human and most other mammals, the sinuatrial valve leaflets have come to lie so far apart that their margins cannot touch. We can assume the competence of the sinuatrial valve is then lost. Previously, it has been suggested that the inability of the sinuatrial valve to guard the right atrium functionally, is the defining feature of anatomical sinus venosus incorporation (Keith & Flack, 1907). In human, after the loss of competence of the sinuatrial valve, the remnants of the right sinuatrial valve leaflet, the Eustachian and Thebesian valve, also known as the venous valves of the heart, and the walls between the sinus horn orifices, including the dorsal wall of the confluence of the sinus horns, have become part of the right atrium (Keith & Flack, 1907). The left sinuatrial valve almost always regresses completely, although some vestiges may be found (Rusu, 2007; Raut et al. 2017). With the sinus venarum defined as the part of the sinus venosus that is visible from within the body of the right atrium, much of the sinus horn myocardium persists outside the right atrium. Therefore, these parts of the sinus horns are not considered to be incorporated into the right atrium (Keith & Flack, 1907).

### Comparative anatomy

Early studies comparing adult vertebrate hearts have revealed that the sinus venosus is proportionally smaller in marsupial and eutherian mammals than in reptiles and amphibians (Keith & Flack, 1907; Benninghoff, 1933). This evolutionary proportional reduction in size has been considered a hallmark feature of the sinus venosus incorporation (Benninghoff, 1933).

When assessing the size of the sinuatrial valve leaflets, many mammals with a sinus venosus that is considered to be incorporated, retain substantial leaflets of the sinuatrial valve. However, these are found to guard the orifice of the inferior caval vein and left superior caval vein only, leaving the right superior caval vein unguarded, as is the case in primates such as the woolly monkey (Rowlatt, 1990). In contrast, adult reptiles and monotreme mammals retain a large sinuatrial valve that appears, by the size of the leaflets, to be able to guard the entire sinuatrial junction functionally, including the ostium of the superior caval vein. Based on this, the sinus venosus of these animals is considered not to be incorporated (Rowlatt, 1990; Jensen et al. 2017). Therefore, a unifying feature of anatomical sinus venosus incorporation, as distilled from the literature, appears to be associated with a smaller sinus venosus and a loss of sinuatrial valve competence around the right superior caval vein.

**Figure 2:**
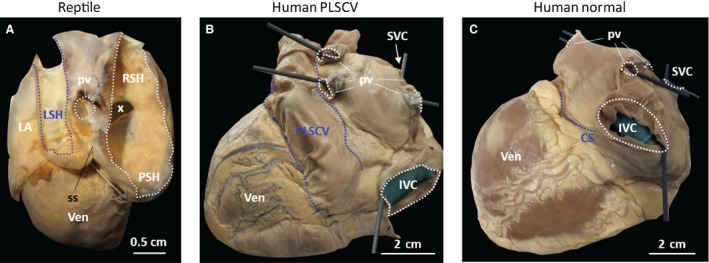
Comparative anatomy of sinus venosus incorporation. When comparing the configuration of the sinus horns, or systemic veins, of reptiles, as exemplified here by the Gila monster (*Heloderma suspectum*) (A), the setting is similar to that of human hearts with a persistent left superior caval vein (PLSCV) (B). The dorsal view of the normal adult human heart (C). Note that the orientation of the hearts corresponds to the orientation they have in the thoracic cavity. ICV, inferior caval vein; LA, left atrium; LSH, left sinus horn; PSH, posterior sinus horn; pv, pulmonary vein; RSH, right sinus horn; SCV, superior caval vein; ss, sinus septum; Ven, ventricle; X, sinuatrial junction.

Some of the literature emphasises an association of the loss of valvar competence with a remodelling of the sinus septum. The sinus septum is not a full septum but a dorsal fold, or ridge, of thicker myocardium between the left sinus horn and the inferior caval vein that, during development, fuses with the right sinuatrial leaflet to form the bridge between the Eustachian and Thebesian valves (Benninghoff, 1933; Steding et al. 1990). In humans, the sinus septum normally remodels to become the roof of the coronary sinus (Steding et al. 1990) and it will become the distal part of the tendon of Todaro (Domènech‐Mateu et al. 1994). In reptiles, the sinus septum does not remodel and will persist in the dorsal wall of the sinus venosus (Jensen et al. 2017), which is revealed by a narrowing of the lumen between the left sinus horn and the remainder of the sinus venosus (Fig. 2A). To see whether valvar competence is related to sinus septum remodelling, one could look at humans with a persistent left superior caval vein (Fig. 2B), a configuration that resembles the reptile setting of the systemic inflow tract. As can be observed in Fig. 2 of Tyrak et al. (2017), the sinuatrial valve is essentially normal and the sinus septum is remodelled, as indicated by the beam of tissue visible between the Eustachian valve and the coronary sinus (Tyrak et al. 2017). However, the specimen with a persistent left superior caval vein that we examined did not show a clear sinus septum, there was no beam between the Eustachian valve and coronary sinus, suggesting that, in this case, the sinus septum may have remodelled to the point that it had been lost. However, the literature is too scant and inconsistent to draw conclusions on sinus septum remodelling as a feature of sinus venosus incorporation across mammalian taxa in general.

### Physiology

Reptiles do not anatomically incorporate the sinus venosus and their sinus venosus contracts before the atria contract (Keith & Flack, 1907; Jensen et al. 2017) (Fig. 1A). The sinus venosus thereby assists in the filling of the right atrium (Jensen et al. 2017). On the ECG, the activation of the sinus venosus is revealed by an SV‐wave. This wave is followed by a delay that ends by the onset of the atrial P‐wave (Mullen, 1967). A sinuatrial delay therefore exists and its duration is comparable to the atrioventricular delay. Like the atrioventricular delay, it, too, is regulated by the vagus nerve and exhibits the Wenckebach phenomenon (Valentinuzzi & Hoff, 1972).

**Figure 3:**
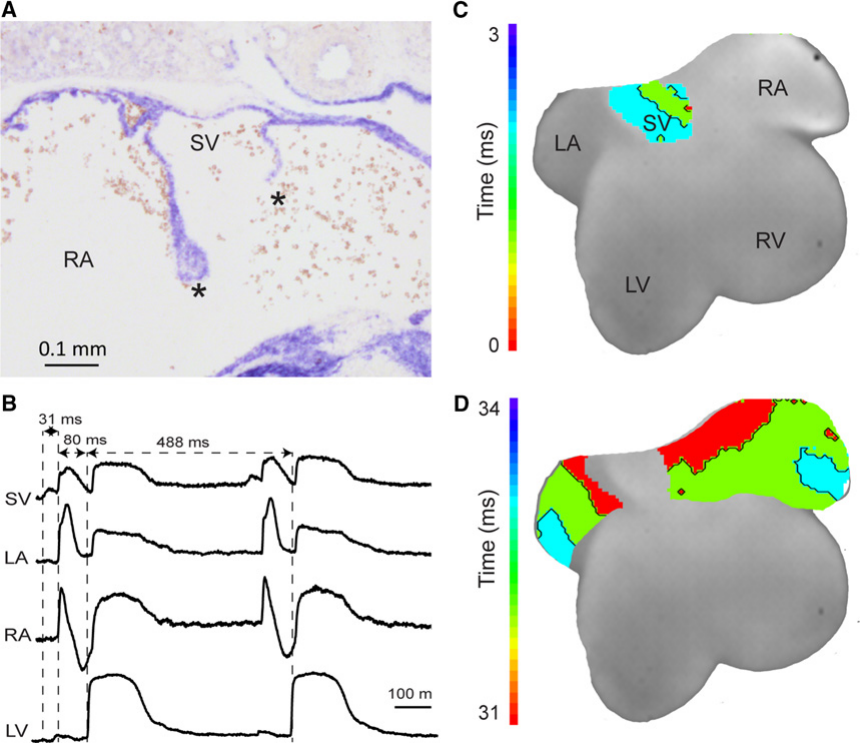
Anatomy and physiology of the embryonic mouse heart. (A) A transverse section of the sinuatrial valve of a mouse of embryonic day 14.5 stained with *in situ* hybridisation for a mix of myocardial RNA probes as described in Soufan et al. (2003). The valve is large enough to potentially guard the entire sinus venosus from the right atrium. Asterisks indicate the sinuatrial valve leaflets. (B) Optically recorded action potentials from the dorsal side of another mouse heart of embryonic day 14.5. The early activation of the sinus venosus (SV), precedes the early atrial activation by 31 ms. (C) Reconstructed activation patterns showing the start of activation in the region of the sinus venosus and activation of the atria after a substantial delay. The delay between sinus venosus and atrial activation is 31 ms on a cardiac cycle of 488 ms. LA, left atrium; LV, left ventricle; RA, right atrium; RV, right ventricle; SV, sinus venosus.

**Figure 4:**
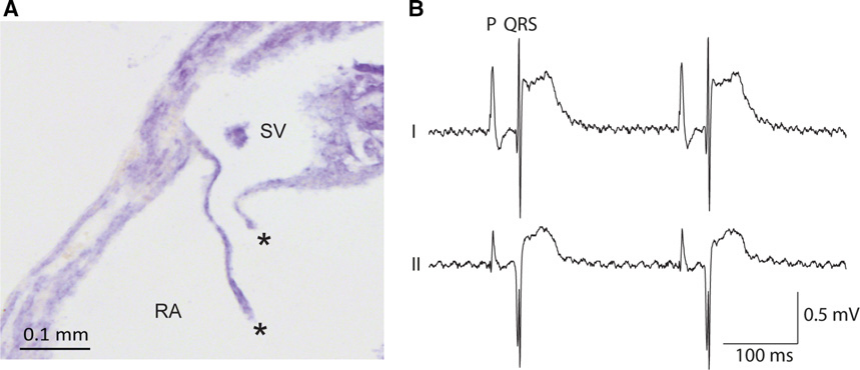
Anatomy and physiology of the neonatal mouse sinuatrial junction. (A) Transverse section of the sinuatrial valve of a 1‐day‐old mouse stained with *in situ* hybridisation for a cTnI RNA probe as described in Aanhaanen et al. (2011). Asterisks indicate the sinuatrial valve leaflets. (B) Electrocardiogram of a mouse of neonatal day 1. Only a P‐wave can be seen before the QRS complex. P, atrial activation wave; QRS, ventricular activation wave; SV, sinus venosus.

**Figure 5:**
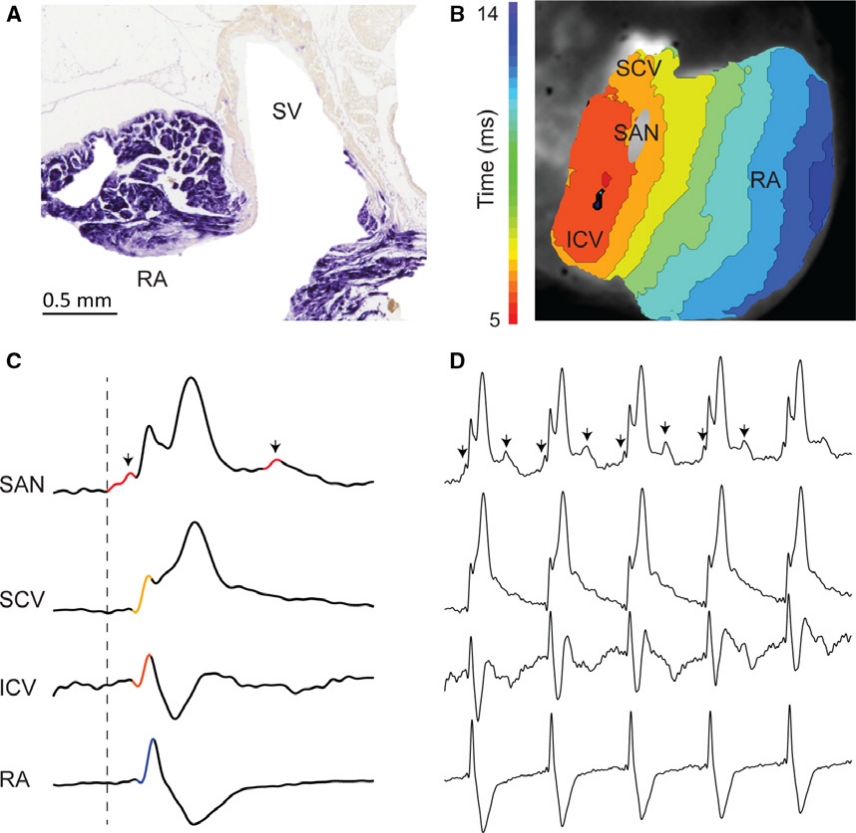
Anatomy and physiology of the adult mouse heart. (A) A transverse section of the sinuatrial valve of an adult mouse stained with *in situ* hybridisation for an *Nppa* RNA probe as described in Hoogaars et al. (2007). There is no evidence of leaflets of the sinuatrial valve that can cover the cavity now known as the sinus venarum (SV). Blood in the cavities has been masked with white. (B) Optical mapping of the epicardial side of the right atrium and intercaval area in the adult mouse. (C) Reconstructed activation patterns show a delay between sinus node activation and working myocardium activation but the caval veins and right atrium activate simultaneously. Colours in the trace also correspond to the colours used in (B). (D) In this case, Under isoproterenol stimulation, only one in two of the sinus node activations (arrows) is propagated to the surrounding working myocardium, suggesting the presence of junctional tissue. ICV, inferior caval vein; RA, right atrium; SAN, sinuatrial node; SCV, superior caval vein; SV, sinus venarum.

Mouse embryos 14.5 days old have not yet anatomically incorporated their sinus venosus, as based on the large size of the sinuatrial valve leaflets that still cover the ostia of all sinus horns (Fig. 3A) (Mommersteeg et al. 2007). Optical mapping of the depolarisation of such an embryonic heart reveals a pattern of depolarisation similar to that in adult reptiles, including a sinuatrial delay (Fig. 3B,C). In contrast to reptiles, however, in postnatal marsupials and eutherian mammals, such as the mouse (Fig. 1B–C), the activation of the sinus myocardium does not precede the activation of the atrial myocardium (Boukens et al. 2018) (Figs 4 and 5). The sinus venosus can therefore be considered to be electrically incorporated into the right atrium (Jensen et al. 2014a, 2017). Regarding the anatomical incorporation of the sinus venosus in mice, it is seen that the right superior caval vein becomes unguarded only in the adult (Fig. 5), not the neonate (Fig. 4 see also the 18.5‐day‐old embryo in Fig. 6 of Bharucha et al. 2015). Likely, the anatomical sinus venosus incorporation is a complementary feature to the loss of sinuatrial delay, but these processes do not necessarily take place at the same time.

**Figure 6:**
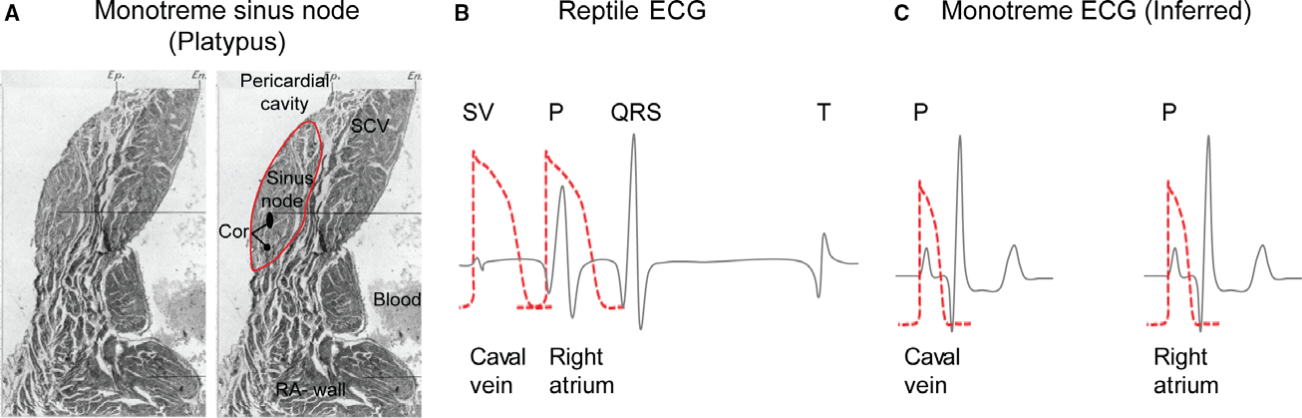
Anatomy and inferred physiology of the platypus heart. (A) Cross‐section of the Platypus (*Ornithorhynchus anatinus*) sinus nodal region, where the sinus node, according to Davies (1931), could be recognised as a region of pale‐stained myocardium (encircled by a red line) receiving a large coronary artery (Cor). The myocardium from the superior caval vein (SCV) to the right atrial wall (RA‐wall) is continuous. Modified from Davies (1931). Compared with the electrical activity of the reptile heart (B), modified from Jensen et al. (2017), the monotreme heart is likely without a sinuatrial delay (C), as the pacemaker myocardium does not form a complete junction between the myocardium of the SCV and right atrium (the electrophysiology of the monotreme is inferred by us, the traces are not based on actual recordings). Cor, coronary artery of the sinus node; P, atrial activation wave; QRS, ventricular activation wave; RA, right atrium; SCV, superior caval vein; SV, sinus venosus activation wave. For methods see previously published literature (Aanhaanen et al. 2011).

We are not aware of electrophysiological studies on monotreme mammals from which the presence or absence of a sinuatrial delay could be assessed. It is noteworthy, however, that adult monotreme mammals do not have a ring of junctional tissue at the entrance to the right atrium like ectotherms (Tessadori et al. 2012; Jensen et al. 2017) but instead have an anatomically distinct sinus node (Davies, 1931; Dowd, 1969). The working myocardium of the sinus venosus and the right atrium can be seen to be continuous adjacent to the sinus node (Fig. 6A). These features likely indicate that monotreme hearts are without a reptilian sinuatrial delay (Fig. 6B,C), which could mean that the sinus venosus is electrically incorporated without being anatomically incorporated due to the presence of the large sinuatrial valve leaflets (Rowlatt, 1990).

### Molecular markers

The gap‐junction protein CX40, encoded for by *GJA5*, allows for fast electrical propagation and is expressed in the myocardium of the atria, pulmonary vein and ventricles in human embryos already before week 5 (Carnegie stage 14) and in mouse embryos at day 9.5 of development (Mommersteeg et al. 2007; Sizarov et al. 2010). In the sinus venosus, *GJA5* is initially not expressed. Also, at this stage, the sinus venosus can be identified by the expression of the transcription factor TBX18, whereas NKX2.5 is absent from it. Furthermore, there is a strong expression of the ion‐channel HCN4, a key ion‐channel in pacemaking (Ludwig et al. 1998; Mommersteeg et al. 2007; Sizarov et al. 2010). By week 6 of human gestation (Carnegie stage 16) or day 14.5 of mouse gestation, the sinus venosus starts to express NKX2.5 and CX40, while HCN4 expression becomes restricted to the future sinus node. This process has been described as ‘atrialisation’ of the sinus venosus (Mommersteeg et al. 2007; Sizarov et al. 2010), even though, as described previously, there may still be an electrical sinuatrial delay present.

In reptiles, a molecular‐defined ‘atrialisation’ also takes place. Their *Tbx18*‐positive sinus venosus will likewise gain the expression of *Nkx2*.*5* and *Gja5* (Jensen et al. 2017). *Hcn4* is in reptiles also expressed strongest in the pacemaker region, which is found at the border of the sinus venosus to the right atrium. Yet, in adult reptiles the sinus venosus remains functionally distinct from the right atrium and is not incorporated. Therefore, it can be concluded that we have not yet found unambiguous molecular makers of sinus venosus incorporation.

### Terminology

The term incorporation implies that the sinus venosus and the atrium become a single structure. However, the sinus venarum, coronary sinus and right superior caval vein (the part containing myocardium) are described as distinct structures on the same hierarchical level in the adult mammalian heart (Rowlatt, 1990; Standring et al. 2016).

The term ‘merger’ (Keith & Flack, 1907; Carmona et al. 2018), according to its etymological meaning of dipping or plunging in (www.etymonline.com/word/merge), implies that the sinus venosus moves to become surrounded by the right atrium. However, this is not true, as much of the sinus venosus myocardium remains in its original position.

With assimilation (D'Cruz & Smith, 1995), the sinus venosus would have to be identical to the atrium, but the expression of, for example, *Tbx18* is not matched by the atrial myocardium.

This leaves atrialisation: becoming more atrial‐like. This term is unambiguous and fits both the anatomical and electrical changes observed in the sinus venosus. However, this term is more widely associated with Ebstein's malformation in which the right ventricle becomes atrialised by the apical displacement of the inferior leaflet of the tricuspid valve (Radford et al. 1985). Therefore, it is more practical to continue using the term incorporation for the changes to the sinus venosus, as that is the most commonly used description.

## Discussion

The incorporation of the sinus venosus has been defined by different criteria that are derived from different lines of evidence that at times use different terminologies. Anatomical observations have been used for more than a century but, as we show, they have not led to a consensus on what constitutes incorporation of the sinus venosus. Instead, the strongest line of evidence may be the functional assessment of features such as the presence of a sinuatrial delay, which relates to the sinus venosus systole that precedes the atrial systole. Unfortunately, precious little data exist on the haemodynamics across the sinuatrial junction in the cardiac cycle. By the use of Doppler echocardiography in the Burmese python, in which the sinus venosus is not incorporated, we have previously shown that flow across the sinuatrial valve has two components, an early passive component and a later active component (Jensen et al. 2014b). This is analogous to the passive and active filling of the ventricles. We are not aware of Doppler echocardiography studies on the sinuatrial junction of mammals, but such studies could settle whether the sinus venosus is incorporated in the setting of a persisting large sinuatrial valve. When the sinuatrial valve is much reduced, such as in the adult human heart, the sinus venosus can be considered to be incorporated.

## Conclusion

We propose that the most operational criterion for sinus venosus incorporation is the loss of a sinuatrial delay which can be, but is not always, accompanied by an anatomical loss of sinuatrial valve competence for at least, the right superior caval vein.

## Conflicts of interest

The authors have no conflicts of interest to disclose.

## Author contributions

J.W.F. and B.J. performed the analysis and drafted the manuscript. B.J.B. acquired the electrophysiological data. J.W.F., B.J.B. and B.J. created the figures. R.J.O. supplied us with the human hearts. All authors reviewed the manuscript and approved it for publication.
